# Effects of HIV-1 protease on cellular functions and their potential applications in antiretroviral therapy

**DOI:** 10.1186/2045-3701-2-32

**Published:** 2012-09-12

**Authors:** Hailiu Yang, Joseph Nkeze, Richard Y Zhao

**Affiliations:** 1 University of Maryland School of Medicine, Baltimore, MD, USA; 2 Department of Pathology, University of Maryland School of Medicine, Baltimore, MD, USA; 3 Department of Microbiology-Immunology, University of Maryland School of Medicine, 10 South Pine Street, MSTF 700, Baltimore, MD, 21201-1192, USA; 4 Institute of Human Virology, University of Maryland School of Medicine, 10 South Pine Street, MSTF 700, Baltimore, MD, 21201-1192, USA

**Keywords:** HIV-1 protease, Structure-based design, Allosteric inhibitor, Antiretroviral therapy, Fission yeast, Cell-based high-throughput screening

## Abstract

Human Immunodeficiency Virus Type 1 (HIV-1) protease inhibitors (PIs) are the most potent class of drugs in antiretroviral therapies. However, viral drug resistance to PIs could emerge rapidly thus reducing the effectiveness of those drugs. Of note, all current FDA-approved PIs are competitive inhibitors, *i.e.*, inhibitors that compete with substrates for the active enzymatic site. This common inhibitory approach increases the likelihood of developing drug resistant HIV-1 strains that are resistant to many or all current PIs. Hence, new PIs that move away from the current target of the active enzymatic site are needed. Specifically, allosteric inhibitors, inhibitors that prohibit PR enzymatic activities through non-competitive binding to PR, should be sought. Another common feature of current PIs is they were all developed based on the structure-based design. Drugs derived from a structure-based strategy may generate target specific and potent inhibitors. However, this type of drug design can only target one site at a time and drugs discovered by this method are often associated with strong side effects such as cellular toxicity, limiting its number of target choices, efficacy, and applicability. In contrast, a cell-based system may provide a useful alternative strategy that can overcome many of the inherited shortcomings associated with structure-based drug designs. For example, allosteric PIs can be sought using a cell-based system without considering the site or mechanism of inhibition. In addition, a cell-based system can eliminate those PIs that have strong cytotoxic effect. Most importantly, a simple, economical, and easy-to-maintained eukaryotic cellular system such as yeast will allow us to search for potential PIs in a large-scaled high throughput screening (HTS) system, thus increasing the chances of success. Based on our many years of experience in using fission yeast as a model system to study HIV-1 Vpr, we propose the use of fission yeast as a possible surrogate system to study the effects of HIV-1 protease on cellular functions and to explore its utility as a HTS system to search for new PIs to battle HIV-1 resistant strains.

## Introduction

HIV/AIDS is one of the most devastating diseases in the world with approximately 34 million people living with HIV in 2010 and approximately 2.7 million new infections in the same year [1]. Use of antiretroviral therapy (ART) can successfully reduce Human Immunodeficiency Virus Type 1 (HIV-1) viral replication, of which HIV-1 protease (PR) inhibitors (PIs) are the most potent viral inhibitors. However, one of the major challenges in using ART is the emergence of viral drug resistance due to mutations in the *PR* gene. Resistant mutations that accumulated during multiple ARTs may lead to cross drug resistance to most or all PIs [2-4], raising a possibility that multi-drug resistant viruses may ultimately outgrow the number of PIs available. Therefore, there is an urgent need to develop new PIs that are active against those drug-resistant HIV-1 PRs (dr-PRs). This review looks at the mechanisms in which HIV-1 PR alters host cellular functions such as apoptosis in CD4^+^ T-lymphocytes, why PIs are such potent drugs, and how a eukaryotic cell-based high-throughput screening (HTS) system using the fission yeast (*Schizosaccharomyces pombe*) as a model organism may accelerate the drug discovery process and prevent dr-PRs from developing.

### Life cycle of HIV-1

**Figure 1:**
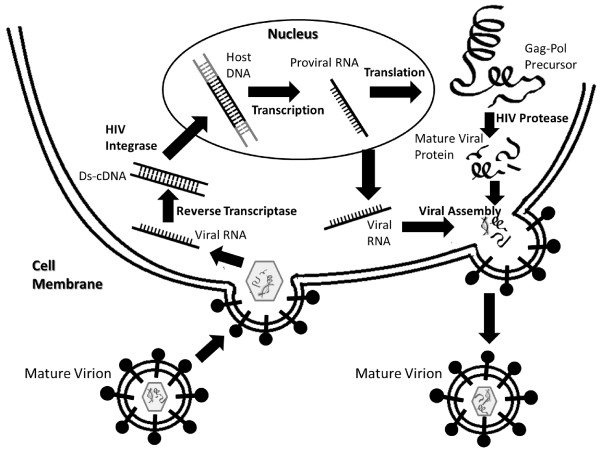
**Life cycle of the HIV-1.** Life cycle of HIV-1 occurs in 6 major steps: 1) adsorption and fusion of viral particle, 2) reverse transcription by Reverse Transcriptase, 3) integration of viral dsDNA into genomic DNA by Integrase, 4) expression of viral genes, 5) cleavage of Gag-pol and Gag precursor by HIV-1 PR and assembly of proteins into mature viral particle, and 6) budding of mature virion from host cell.

The life cycle of HIV-1 (Figure 1) comprises of the following distinct stages: 1) adsorption and fusion, 2) reverse transcription, 3) integration, 4) viral gene expression, 5) virus assembly and maturation, and 6) budding. The matured infections virion consists of two copies of genomic RNA and functional viral proteins: reverse transcriptase (RT), integrase (IN) and protease (PR). When the HIV −1 virion is uncoated into the targeted host cell such as a CD4^+^ T-lymphocyte, RT catalyzes the conversion of one copy of the genomic viral RNA into a double-stranded viral DNA (dsDNA) [5]. IN catalyzes its integration into the host chromosome to form a proviral DNA [5-8]. Using the host cellular system, copies of HIV-1 genomic material as well as shorter strands of messenger RNA (mRNA) are created. The mRNA strands are used as blueprint to make long chains of HIV-1 precursor proteins. The precursors are then cut by HIV-1 PR into smaller active proteins and assembled into mature virions. Following the assembly of viral RNA strands and smaller active proteins into a new viral particle, the virion buds from the host cell to infect another cell [9,10].

During the virion assembly phase of HIV-1 replication, HIV-1 PR performs a series of 12 cleavages on the Gag, Gag-Pol and Nef polyprotein precursors [11-15]. These cleavages proceed in a sequential and highly specific manner to produce active viral enzymes (RT, PR and IN), viral structural proteins (capsid and nucleocaspid), and other viral factors essential for viral replication and infection [11,14]. Despite the specificity and sequential manner of these cleavages, the 12 proteolytic sites bear little resemblance to each other. There is currently the real substrate of HIV-1 PR and the factors governing substrate recognition [11,12,16].

### HIV-1 protease

**Figure 2:**
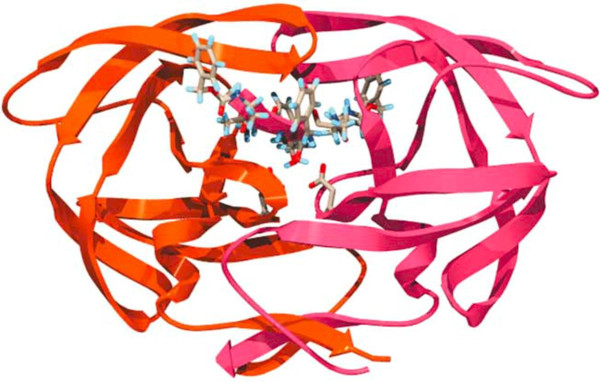
**Structure of HIV-1 protease bound to TL-3, a competitive protease inhibitor**. Figure was obtained with permission from [9].

PR belongs to the family of aspartic proteases. The structure of PR (Figure 2) is a homodimer and consists of subunits of 99 amino acid residues [5,9,17-22]. Each subunit is made up of nine β-strands and a single α-helix. Four anti-parallel β-strands form the highly stable dimer interface which constitutes the active site [5,19,20,22,23]. The core of the active site is hydrophobic and contains two aspartic acid residues contributed by both subunits. Flexible anti-parallel β-sheets from both monomers form two flaps that cover the active site thereby restricting access to it [5,21,24-26]. In the free enzyme state, the flaps assume a semi-open conformation [21,22,27,28] and with a ligand in the active site, they assume a closed conformation [5,22,26,29-31]. It has been reported that, a network of weakly polar interactions between the flaps keeps them in a semi-open conformation [21]. Two models have been used to explain the mechanisms of flap opening and closing. In the first model, the ligand forms a collision complex with HIV-1 PR in the open flap conformation as it enters the active site of the PR and then induces the flaps to close [32]. In the second model, the ligand approaches the HIV-1 PR in the semi-open flap conformation and then induces the flaps to adopt an open conformation as it enters the active site. The flaps then extend over the substrate and allow proteolysis to occur [21,32,33]. The PR cleavage site on the Gag and Gag-Pol precursor contain the unique amino acid sequences of Phe-Pro and Tyr-Pro [5].

### Effects of HIV-1 PR on host cellular functions

In addition to cleaving viral precursors, HIV-1 PR also cleaves host cell proteins. There is growing evidence that proteolysis and depletion of cellular proteins lead to both necrotic cell death and apoptotic cell death of the infected CD4^+^ T-cell. This can occur through several pathways and contribute to the overall CD4^+^ T-cell depletion in HIV/AIDS patients.

HIV-1 infection leads to necrotic/lytic cell death of the infected CD4^+^ T-cell, resulting in CD4^+^ T-cell depletion, a hallmark of HIV/AIDS [34,35]. However, the specific mechanisms of CD4^+^ T-cell depletion is elusive. Recently, CD4^+^ T-cell depletion has been directly associated with the actions of HIV-1 PR. Blanco et al. found that HIV-1 *PR* expression in COS7 monkey kidney cells resulted to visual changes associated with cell necrosis such as accumulation of cell debris, cellular swelling, vacuolization, and loss of plasma membrane integrity [36]. Treatment of HIV-1 *PR* expressing C8166 human lymphocytes and COS7 cells with the protease inhibitor Saquinavir inhibited these necrotic effects [36,37]. Furthermore, removal of Saquinavir from *PR*-expressing C8166 cells resulted to reactivation of HIV-1 PR and cellular necrosis [37]. These results suggest that the necrotic process is a direct result of the proteolysis of cellular proteins by HIV-1 PR. The exact mechanism(s) leading to necrotic cell death is currently unclear.

HIV-1 PR proteolysis of cytoskeletal proteins has been linked to necrotic or apoptotic cell death. HIV-1 PR has been shown *in vitro* to cleave many cytoskeletal proteins, including actin, desmin, myosin, tropomyosin, troponin C, vimentin, alzheimer amyloid precursor protein, and glial fibrillary acidic protein [38-43]. Of these cytoskeletal proteins, vimentin is a known substrate for HIV-1 PR *in vivo*. Injection of purified HIV-1 PR into human fibroblastic cells resulted to in disruption of stress fibers, collapse of the cytoplasmic vimentin intermediate filaments, and changes in nuclear morphology and chromatin organization [43]. The cytoplasmic structural changes are a direct result of cleavage of cytoplasmic vimentin and other structural proteins by HIV-1 PR [43]. Changes in nuclear morphology and chromatin organization are believed to be initiated by an N-terminal fragment of cleaved vimentin [39]. When HIV-1 PR cleaves vimentin, it produces an N-terminal fragment that, unlike uncleaved vimentin, is able to infiltrate the nucleus and cause structural changes *via* a mechanism currently unclear [39]. These cellular effects are certainly detrimental and likely to be involved in either necrosis or apoptosis. However, there is currently inadequate evidence to support whether cleavage of these cytoskeletal proteins triggers cell death and if so, how.

HIV-1 PR induces CD4^+^ T-cell apoptosis by decreasing concentration of cellular protein Bcl-2 [44,45], an anti-apoptotic member of the Bcl-2 protein family [46]. Strack et al. found that prior to apoptosis in several cell lines induced to express HIV-1 *PR*, intact Bcl-2 was undetectable and fragmented Bcl-2 level was abnormally high [44]. Further experiments showed that ectopic expression of *bcl-2* in *PR*-expressing lymphocytes *in vivo* and *in vitro* decreased apoptosis and suppressed HIV-1 PR activity, indicating that Bcl-2 protects cells from the cytotoxic effects of HIV-1 PR and apoptosis [44]. Additionally, cells expressing *bcl-2 in vitro* and *in vivo* showed lower rates of apoptosis compared to cells that did not, suggesting that Bcl-2 depletion is a requirement for PR-induced apoptosis [44]. The loss of anti-apoptotic function of the cleaved Bcl-2 is likely due to removal of the BH3 and BH4 domain following cleavage between residue 112 and 113 [44,47]. Normally, Bcl-2 inhibits apoptosis by dimerizing with pro-apoptotic factors of the Bcl-2 protein family. Both BH3 (ligand domain) and BH4 (cell death protecting domain) are essential for this function: BH3 is responsible for binding to BH3 containing pro-apoptotic factors [48] and BH4 is responsible for interacting with Raf kinases [47,49]. Hence, removal of these domains will most likely result to a loss of Bcl-2 function, leading to apoptosis.

HIV-1 PR also induces apoptotic cell death *via* the proteolysis of Procaspase 8 between residue 355 and 356 to form Casp8p41, a truncated form of Procaspase 8 that signals cell death [50-52]. The exact mechanism by which Casp8p41 causes apoptosis has not been elucidated, but several key players have been identified. First, cleavage of Procaspase 8 into Casp8p41 is essential for this apoptosis-inducing pathway. When HIV-1 *PR* is transfected into I.9.2 cells, a T-lymphocyte cell line producing cleavage-resistant Procaspase 8, apoptosis is drastically reduced compared to cells producing *wild-type* Procaspase 8 [52]. Second, Casp8p41 acts through the intrinsic/mitochondrial apoptotic pathway, a pathway in which internal stimuli induce mitochondrial release of pro-apoptotic proteins to carry out apoptosis. Casp8p41 localizes in the mitochondria, the initiation site of the intrinsic apoptotic pathway [53]. In addition, Casp8p41 pathway requires Caspase 9 and Bax/Bak; *casp8p41* transfection in cells with *caspase 9* or *bax/bak* knockout causes minimal cell death compared to non-knockout cells [53]. Caspase 9 is an initiator caspase of the intrinsic apoptotic pathway that activates Procaspase 3 into Caspase 3, the most important executioner caspase [46]. Bax and Bak are both pro-apoptotic members of the Bcl-2 protein family that govern mitochondrial membrane permeability [46], which activates the intrinsic apoptotic pathway with Bax and Bak being essential regulators. Evidence suggests that the Casp8p41 pathway is a major cause of cell death associated with HIV-1 PR. Lymphoid tissues from HIV-1 infected patients showed that cells with Casp8p41, experienced a drastically increased rate of apoptosis and higher levels of pro-apoptotic factor Caspase 3 compared to cells void of Casp8p41 [50,52]. Furthermore, inhibition of HIV-1 PR cleavage of Procaspase 8 into Casp8p41 in I.9.2 cells (described above), resulted to in a large reduction of cell death in cells transfected with HIV-1 *PR*[52].

It is clear that HIV-1 PR’s role is not limited to the cleavage of viral precursor proteins and assembly of the mature virions. HIV-1 PR cleaves an array of cellular proteins and contributes to HIV-induced cytotoxicity through several pathways.

### Current state of HIV therapy

Currently, the gold standard of HIV treatment is highly active antiretroviral therapy (HAART). HAART is a cocktail of drugs from several classes of antiretroviral drugs (ARVs), each of which inhibits one step of the HIV-1 life cycle. For example, PIs prevent protease from cleaving the Gag and Gag-Pol precursor polyproteins, thus inhibiting the assembly and maturation of the viral particle; RT inhibitors prevent conversion of viral genomic RNA into viral DNA, thus stopping viral DNA integration into host chromosome. The multiple ARVs present in HAART work together to suppress HIV-1 at multiple stages of the viral life cycle. For many years, HAART has been proven to effectively decrease viral load and increase CD4^+^ T-cell count, thus improving the quality of life of HIV/AIDS patients. Recent reports show that a HIV-1 infected patient can expect to have a close-to-normal life span if the virus is diagnosed early and treated with adequate HAART [54,55].

### HIV-1 protease inhibitors

Of all the ARV drugs, PIs are the most potent because they improve the clinical outcome of HIV patients in three important ways. First, PIs suppress HIV-1 PR by preventing the assembly of mature virion, thus preventing viral replication and decreasing viral load. Second, PIs inhibit PR cleavage of host cell proteins (described above), which reduces PR-related cytotoxicity, apoptosis, and necrosis in infected host cells. Third, PIs have anti-apoptotic effects *via* a mechanism not directly related to HIV-1 PR suppression. This emerging area of research will not be discussed in this paper, but is extensively reviewed in [56,57]. These three major benefits of PIs make them essential to any HAART.

Because PIs are so important to HAART, the development of viral resistance to PIs may have dire public health consequences. Currently, there are eleven FDA approved PIs on the market [58]. These PIs all belong to the same mechanistic class of competitive inhibitors: inhibitors that bind to the active site of PR to prevent substrate association [59,60]. One major problem with this commonality among PIs is that *PR* mutations in the active site may interfere with active site geometry and lead to cross-resistance to multiple or all protease inhibitors [2-4,61]. To combat this problem, we need to consider potential alternative strategies such as developing allosteric PIs, *i.e.,* non-competitive inhibitors that inhibit HIV-1 PR at a site other than the active site. The existing *PR* mutants resistant to the competitive PIs are less likely to confer cross-resistance to the allosteric PIs because allosteric inhibitors do not compete with substrates for the active site.

One class of promising allosteric inhibitor is the compounds that interact with the flaps covering the HIV-1 PR active site (Figure 2). Alteration of the flaps interferes with substrate docking to reduce PR activity. Polyoxometalates (POM) are a class of large inorganic flap-binding allosteric inhibitors [62]. Although POMs are potent PIs, they were initially found to be too toxic for clinical use [63]. Recent research has been focused on modifying the functional groups of POMs in a way that maintains its potency while reducing its cytotoxicity [64]. A particularly promising group of candidate PIs are the C3-substituted cyclopentyltetrahydrofuranyl (Cp-THF) compounds, a recently designed group of flap-binding small molecule inhibitors [65,66]. Ghosh et al. found that several Cp-THFs effectively inhibit a panel of dr-PRs *in vitro*, demonstrating their potential to combat dr-PRs [65,66].

Another allosteric PI class being investigated are dimerization inhibitors. Dimerization inhibitors inhibit HIV-1 PR by binding to the dimerization interface and preventing the formation of the active PR homodimer. Bouras et al. first reported a dimerization inhibitor that interfered with the β-sheet dimerization interface of the HIV-1 PR monomer [67]. Chmielewski et al. have also been developing a dimerization inhibitor design [68-72] that cross-links the interfacial peptides of HIV-1 PR to prevent formation of the active homodimer protein [71].

Significant progress has been made towards designing allosteric inhibitors, but we currently have no FDA-approved allosteric inhibitor. Like current FDA-approved competitive inhibitors, all allosteric inhibitors in development were discovered through a structure-based design. In a structure-based design, inhibitors are selected, using structural data or computational predictions, to fit a specified site on the target protein and assayed *in vitro* for binding affinity. The inhibitors are then improved upon by a trial-and-error process in which structural modifications are made, assayed for binding affinity, and compared. After the *in vitro* potency of the drug has been demonstrated, the drug is then tested *in vivo*. There are several limitations to this system. The structure-based method requires extensive knowledge of the structure of the substrate and inhibitor, limiting what it can screen for. Since a structure-based design focuses on fitting an inhibitor to a specific site on the protein (usually the active site), it can only look for inhibitors to one site at a time. Finally, the structural design may generate PIs that are target specific and potent, but come with strong adverse effect such as cellular toxicity, thus diminishing their efficacy and applicability.

### Cell-based drug screenings and future perspectives

A cell-based system to screen for PIs holds several advantages over a structure-based design. First, since a cell-based system does not require structural knowledge of the target protein, it is not restricted by the availability of structural data of proteins such as dr-PRs with conformational changes from the wild-type PR. Second, allosteric PIs could be sought in a cell-based system purely based on their inhibitory effect of HIV-1 PR-induced cell death without considering the site of inhibition. This is because a cell-based HTS looks at results at the cellular level, allowing it to target the whole HIV-1 protease. What this means is that a cell-based system can potentially identify all types of PIs at once, regardless of their binding site or mechanism of action. The third distinctive advantage of a cell-based system over structure-based designs is that a cell-based system can eliminate any compounds that have strong cytotoxic effect. Most importantly, a simple, economical, and easy-to-maintained cellular system will allow searching for potential PIs in a large-scaled HTS system, increasing our chances of success. Thus, cell-based drug screening systems may be a useful alternative strategy that will overcome many of the inherited shortcomings associated with the structure-based drug design and identify new PIs that would be difficult to develop using structure-based designs.

There are currently several cell-based PI screening systems using *Escherichia coli (E. coli)*[73,74] or human cells [75-77]. All of these systems are capable of screening for HIV-1 PR activity and two of them also are capable of screening for cytotoxicity [74,76]. Cheng et al. developed a cell-based assay model for PR-induced cytotoxicity by expressing HIV-1 *PR* in *E. coli*[74], an organism that shows cytotoxicity upon *PR* expression, just like in mammalian cells [78,79]. Fuse et al. expressed HIV-1 *PR* in a chimeric protein along with green fluorescence protein in a novel human kidney cell line (E-PR293) to assay for both PR levels and cytotoxicity [76]. These systems are useful, but neither is ideal for cell-based HTS. *E. coli* is a prokaryotic organism that is different from a mammalian cell in many ways, so results obtained from *E. coli* may not translate well to humans. E-PR293 is a human cell line, which is expensive and time-consuming to maintain. Hence, the system developed by Fuse at al. will make a good confirmatory assay, but may be too expensive for cell-based HTS. Therefore, we are still in need of a suitable model organism for a viable cell-based HTS system.

An ideal model organism for PR studies should: 1) be easy to maintain and manipulate, 2) share fundamental cellular features and processes with mammalian cells, and 3) have cellular response to PR similar to cellular effects of PR in mammalian cells. Fission yeast possesses many of these properties. Specifically, fission yeast is a unicellular organism that is very easy to grow and manipulate in the laboratory. It typically divides every 3–4 hours at 30°C with active agitation compared to 24 hours for human cell lines. Despite its simplicity, fission yeast, as a eukaryotic organism, shares very similar fundamental cellular features and processes as mammalian cells. For example, it has similar cell cycles as cells of higher eukaryotes [80,81]. In addition, fission yeast contains a splicing mechanism that is able to remove introns from genes of higher eukaryotes and mammals [81-83]. Since fission yeast is capable of post-translational modification (*e.g.,* phosphorylation and acetylation), the heterogeneous proteins produced by fission yeast are very close to their natural forms in mammalian cells [80,82,83].

Based on the above mentioned properties, a fission yeast cell-based HTS system may be a feasible alternative for future screenings of PIs. Its feasibility is supported by the fact that fission yeast has been used extensively as a model system to study HIV-1 viral protein R (Vpr) during HIV-1 infection {reviewed in [84-86]}. For instance, Vpr induces similar cellular changes in both fission yeast and human cells [87], which include: 1) cell cycle G2/M arrest [86,88,89], 2) cytoplasm to nuclear transport of viral pre-intergration complex [81,89], and 3) induction cell death and apoptosis [88-90]. Taking advantage of Vpr-induced cell cycle arrest and cell killing in fission yeast, Benko et al. has established a HTS system for screening HIV-1 inhibitors and cellular suppressors. In a pilot study, they have shown that this cell-based HTS is capable of picking out a Vpr inhibitor from a chemical drug library [91]. This HTS system is currently being employed by the National Institutes of Health Chemical Genomics Center to screen for Vpr inhibitors.

As for the cellular effects of HIV-1 PR, there is currently no published data on whether HIV-1 PR behaves similarly in fission yeast and in mammalian cells. However, based on our many years’ experience in studying HIV-1 Vpr in fission yeast cells, we have reason to believe that fission yeast should be a reasonable model to study HIV-1 PR as well. Specifically, since expression of HIV-1 *Vpr* induces cell death *via* apoptosis in both fission yeast and mammalian cells [86-90], it is likely that expression of HIV-1 *PR*, which also causes cell death and apoptosis in mammalian cells, may have a similar effects in fission yeast and mammalian cells. Blanco et al. showed that expression of HIV-1 *PR* in budding yeast (*Saccharomyces cerevisiae*) causes cell death [36], further supporting the idea that HIV-1 *PR* expression should have a similar effect in fission yeast.

It should be mentioned that although fission yeast may serve as a reasonably good model organism for HTS of PIs, it certainly has its own limitations. For example, fission yeast cells have cell walls but human cells do not, which may limit input of large molecule into the cell. In addition, fission yeast cells only have 3 chromosomes while human cells have 23 pairs. Hence, while results obtained from studying PR and PI in fission yeast are more translatable to humans than many organisms such as *E. coli*, confirmatory test in human cell system are still needed to validate their clinical efficacy.

Our current and future effort will focus on the studying and characterization of HIV-1 PR in fission yeast and to further explore its utility as a HTS system to search for new PIs.

## Conclusion

In this review, we have shown that HIV-1 PR cleaves viral proteins as well as host cell proteins. The cleavage of host cell proteins results in cell death *via* several necrotic and apoptotic pathways, possibly leading to depletion of CD4^+^ T-cells. Thus, PIs are particularly useful as ARVs not only because they inhibit viral replication but also because they rescue host immune cells. One of the downsides of current PIs is the rapid emergence of drug resistance due in part to the fact that all current PIs were designed against the same site on HIV-1 PR: the enzymatic site. To combat drug resistance and avoid multi-drug resistance, allosteric PIs are needed. There are currently several promising allosteric PI candidates. However, development of those drugs has been slow because of the inherited limitations of their structure-based design. Alternatively, cell-based drug screening system could potentially be a good alternative approach that is not limited by the systematic shortcomings of the structure-based design. However, the current challenge associated with using a cell-based system is that there is no suitable model organism to carry out this assay in an economically feasible way. Fission yeast might be a good alternative because it is a single-cell eukaryotic organism. Hence, it is easy to maintain and manipulate in the laboratory, amendable for large-scale HTS, and most importantly, it shares many fundamental cellular features and processes with mammalian cells. Thus we propose that future efforts should focus on studying and characterizing HIV-1 PR in fission yeast and further exploring its potential application as a HTS system for new PIs.

## Abbreviations

ARV: Anti-retroviral drug; Cp-THF: C3-substituted cyclopentyltetrahydrofuranyl; dr-PR: Drug resistant HIV-1 protease; ds-DNA: Double stranded DNA; FDA: Food and Drug Administration; HAART: Highly active antiretroviral therapy; HIV–1: Human Immunodeficiency Virus Type-1; HTS: High throughput screening; IN: Integrase; mRNA: Messenger RNA; PI: Protease inhibitor; POM: Polyoxometalates; PR: Protease; RT: Reverse transcriptase; Vpr: Viral protein R.

## Competing interest

The authors declare that they have no competing interests.

## Authors’ contribution

HY designed and drafted the manuscript. JN drafted part of the manuscript and helped to revise the manuscript. RZ designed and revised the manuscript. All authors read and approved the final manuscript.
